# Performance and comparison of artificial intelligence and human experts in the detection and classification of colonic polyps

**DOI:** 10.1186/s12876-022-02605-2

**Published:** 2022-12-13

**Authors:** Ming-De Li, Ze-Rong Huang, Quan-Yuan Shan, Shu-Ling Chen, Ning Zhang, Hang-Tong Hu, Wei Wang

**Affiliations:** 1 Department of Medical Ultrasonics, Ultrasomics Artificial Intelligence X-Lab, Institute of Diagnostic and Interventional Ultrasound, The First Affiliated Hospital of Sun Yat-Sen University, 58 Zhongshan Road 2, Guangzhou, 510080 People’s Republic of China; 2 Department of Gastroenterology, The First Affiliated Hospital of Sun Yat-Sen University, Guangzhou, China

**Keywords:** Colonic polyps, Endoscope, Artificial intelligence, Deep learning

## Abstract

**Objective:**

The main aim of this study was to analyze the performance of different artificial intelligence (AI) models in endoscopic colonic polyp detection and classification and compare them with doctors with different experience.

**Methods:**

We searched the studies on Colonoscopy, Colonic Polyps, Artificial Intelligence, Machine Learning, and Deep Learning published before May 2020 in PubMed, EMBASE, Cochrane, and the citation index of the conference proceedings. The quality of studies was assessed using the QUADAS-2 table of diagnostic test quality evaluation criteria. The random-effects model was calculated using Meta-DISC 1.4 and RevMan 5.3.

**Results:**

A total of 16 studies were included for meta-analysis. Only one study (1/16) presented externally validated results. The area under the curve (AUC) of AI group, expert group and non-expert group for detection and classification of colonic polyps were 0.940, 0.918, and 0.871, respectively. AI group had slightly lower pooled specificity than the expert group (79% vs. 86%, *P* < 0.05), but the pooled sensitivity was higher than the expert group (88% vs. 80%, *P* < 0.05). While the non-experts had less pooled specificity in polyp recognition than the experts (81% vs. 86%, *P* < 0.05), and higher pooled sensitivity than the experts (85% vs. 80%, *P* < 0.05).

**Conclusion:**

The performance of AI in polyp detection and classification is similar to that of human experts, with high sensitivity and moderate specificity. Different tasks may have an impact on the performance of deep learning models and human experts, especially in terms of sensitivity and specificity.

## Introduction

Colorectal cancer (CRC) is one of the most common malignant tumors in the world and the fourth leading cause of cancer death [1]. Most colorectal cancers are adenocarcinomas that develop from adenomatous polyps [2]. Colonoscopy is the gold standard for screening CRC [3]. Adenoma detection rate (ADR) is the quality index of colonoscopy [4], which is closely related to the prognosis of colon cancer. When ADR increased by 1.0%, the incidence of colorectal cancer decreased by 3.0% [5, 6]. There are two factors that affect ADR: one is visual blindness, and the other is human error. The research results of Ana Ignjatovic et al. [7] showed that doctors with different experience had significant differences in the accuracy of polyp identification (*P* < 0.001). Blind areas of visual field can be solved through the upgrading of instruments [4], while human errors depend on the proficiency of endoscopic surgeons in operating skills. Studies showed that 22–28% of patients who undergo colonoscopies had missed diagnosis of polyps [8, 9], which may lead to advanced diagnosis of colon cancer. How to detect polyps early and classify them accurately is the key to reduce colorectal cancer [10].

Artificial intelligence (AI), a general term for computer programs that simulate human cognitive functions such as learning and problem solving, shows a more stable ability to diagnose micro-adenomatous polyps [11, 12], including traditional machine learning (ML) and deep learning (DL) [13]. Therefore, artificial intelligence may be a solution to reduce the rate of missed diagnosis of polyps and improve the ability of detection [14]. ML uses specific characteristics, such as polyp size, shape, and mucosal patterns, to build descriptive or predictive models [15]. However, these feature patterns, such as edge shape and context information, are often similar in the normal structure of polyp and polyp-like, which reduces the model performance for detection [14]. DL is a network model based on the structure of human brain neural system, especially convolution neural network (CNN). It relies on convolution kernel to extract features from image. Through weight sharing and extraction of local features and semantic information, CNN can reduce the error between predicted values and actual results, which may be some reasons for good performance of CNN in detection and classification [15]. In the Medical Image Computing and Computer Assisted Intervention Society (MICCAI) 2015 polyp detection challenge, the performance of the CNN-based method was better than manual features-based method [16]. Several studies have proved the feasibility of using artificial intelligence to classify colorectal polyps, and exciting results have been obtained [11, 17–20]. Ana Ignjatovic et al. [7] showed that with the assistance of AI, the accuracy of doctors at all stages had been significantly improved (*P* < 0.001).

Studies have shown that AI is different from human doctors in the diagnosis of colon polyps, depending on the experience level of human doctors. Gross et al. [17] compared the diagnostic performance of 2 experts, 2 non-experts, and a computer-based algorithm for polyp classification. The results showed that the sensitivity (93.4%, 95.0% vs. 86.0%, *P* < 0.001), accuracy (92.7%, 93.1% vs. 86.8%, *P* < 0.001) and negative predictive values (90.5%, 92.4% vs. 81.1%, *P* < 0.001) of expert group and AI were significantly better than those of non-expert group. Chen et al. [21] compared the accuracy of the diminutive polyp classification of humans with AI. The results showed that the diagnostic performance of AI (NPV > 90%) met the “leave in situ” criteria proposed by the Preservation and Incorporation of Valuable Endoscopic Innovations (PIVI) initiatives, however, the diagnostic abilities of non-experts (NPV < 90%) were not satisfactory. At the same time, the speed of AI diagnosis is significantly faster than that of experts and non-experts (*P* < 0.001). Misawa et al. [22] compared the diagnostic ability of AI, four experts, and three non-experts. The result showed that overall diagnostic accuracy of AI was higher than that of non-experts (87.8 vs. 63.4%; *P* = 0.01), but similar to experts (87.8 vs. 84.2%; *P* = 0.76), however, AI (94.3%) was superior to both human experts (85.6%, *P* = 0.006) and non-experts (61.5%, *P* < 0.001) in the direction of sensitivity.

Although AI can generally reach the level of human experts, in different studies, the diagnostic performance of AI varies greatly from that of doctors with different experience. At the same time, there were few review studies on the diagnosis of colon polyps between AI and human endoscopic doctors. Therefore, it is necessary to analyze them, so as to better guide the application of AI in clinical practice. The main purpose of this study is to analyze the performance of different AI models in endoscopic colonic polyp detection and classification and to compare them with doctors with different experience.

## Material and method

### Literature search

In this analysis, PubMed, EMBASE, Cochrane, and conference proceedings citation index were searched. The literature retrieval time was up to May 2020, and the language was limited to English. We used “Colonoscopy”, “Colonic Polyps”, “Artificial Intelligence”, “Machine Learning”, “Deep Learning”, “Neural Networks”, “computer-assisted” as the retrieval theme word. A manual search is conducted for the bibliography, citations, and related articles included in the study to search for any other relevant articles that may be missing.

### Inclusion and exclusion criteria

The inclusion criteria for relevant studies were as follows: (1) Research on artificial intelligence in colonic polyp detection/diagnosis. (2) document provides the detailed data to construct diagnose 2 * 2 contingency table. Studies were excluded if duplicate articles or if they were meeting abstracts, reviews, comments, case reports or descriptive studies.

### Data selection and extraction

The two evaluators, (LMD, HHT), independently screened the literature according to the inclusion and exclusion criteria and extracted the data included in the literature. If there was a disagreement, it would be decided by discussion. The relevant inclusion and exclusion criteria for each included studies were showed in Table 2. According to the results of the included studies, we extracted binary diagnostic data (including true positive (TP), false positive (FP), true negative (TN) and false negative (FN)) under corresponding report thresholds and confusion matrix. If the same research contains more than one contingency table, pooled data of each table were used for comparison of results [17]. The following data were also extracted from each study: Author name, title, year of publication, country, sample size, type of AI, number of endoscopic physicians, and external validation. These data are summarized in Tables 1 and 3. According to the included studies, here, the expert is defined as a gastroenterologist with 4–8 years or more on experience performing colonoscopy or 200–1000 colonoscopies, and novice is defined as a gastroenterologist with 0–4 years or less of experience performing colonoscopy or 0–200 colonoscopies [7, 21, 23, 24].

**Table 1:** Characteristics and results of the eligible studies

Author	Years	Main purpose	Model type	Image modality; magnified (if any)	Train set	Test set	Different seniority; endoscopists	External validation
Halligan [24]	2006	Detection	CAD	CT colonography	239 patients	110 patients	No; 10 experts	No
Petrick [38]	2008	Detection	CAD system	CT colonography	UC	UC	No; 4 experts	No
Tischendorf [39]	2010	Classification	Linear classifier、K-NN*、SVM*	NBI; × 100	UC	UC	No; 2 experts	No
Ignjatovic [7]	2011	Classification	UC	NBI	UC	30 polyps	Yes; 2 experts, 1 novice	No
Gross [17]	2011	Classification	SVM	NBI; × 150	NA	434 polyps	Yes; 2 experts, 2 novices	No
Mang [40]	2012	Detection	CTC CAD system	CT colonography	UC	UC	No; 0	No
Mesejo [23]	2016	Classification	RF*、RS*、SVM	WL, NBI	UC	UC	Yes; 1 expert, 1 novice	No
Mori [41]	2018	Classification	SVM	NBI, methylene blue staining; × 520	UC	UC	No; 0	No
Renner [42]	2018	Classification	CNN*	WL, NBI;@@@Without magnification	602 images	186 images	No; 8 experts	No
Chen [21]	2018	Classification	DNN-CAD*	NBI; @@@Optical maximum magnification	2157 images	284 images	Yes; 2 experts, 4 novices	No
Shin [43]	2018	Detection	SVM	UC	UC	UC	No; 0	No
Byrne [11]	2019	Classification	CNN	NBI	223 videos, @@@60,089 images	125 videos	No; 0	No
Cristina [44]	2019	Classification	SVM	WL, NBI; @@@Without magnification	UC	UC	No; 2 experts	No
Zachariah [35]	2020	Classification	CNN	WL, NBI	5278 images	634 images	No; 0	Yes
Shahidi [45]	2020	Classification	CNN	WL, NBI, near-focus	UC	UC	No; 0	No
Qadir [14]	2020	Detection	Faster R-CNN	UC	UC	UC	No; 0	No

CT colonography, computed tomographic colonography; NBI, narrow-band imaging; WL, white light; CAD, computer-aided diagnosis; K-NN, k-nearest neighbor; RF, random forests; RS, random subspaces; SVM, support vector machine; CNN, convolutional neural network; DNN-CAD, computer-aided diagnosis with a deep neural network; UC, unclear

### Quality assessment

The quality grading of the literature was determined by the Quality Assessment of Diagnostic Accuracy Studies (QUADAS-2) guidelines. The QUADAS-2 includes four parts regarding patient selection, index test, reference standard and flow and timing of risk of bias. The risk of bias was classified as ‘low’, ‘high’ or ‘unclear’ [25, 26]. The evaluation was conducted by two reviewers (LMD, HHT) independently, and the evaluation nonconforming was decided by discussion.

### Statistical analysis

We examined the heterogeneity of the included literature. Heterogeneity among the studies included in the meta-analysis was assessed using Cochran’s Q test. Random effects model using Der Simonian and Laird method was considered when heterogeneity was found [27]. Furthermore, we calculated the pooled sensitivity (S_EN_), specificity (S_PE_) and 95% confidence interval (CI) of each study. Then, we plot the summary receiver operating characteristic curve (sROC), and calculate the area under the curve (AUC). The 95% CI of the sensitivity and specificity were compared between different subgroups. Non-overlapping 95% CIs between 2 subgroups were used to define statistically significant difference (*P* < 0.05) [12]. Statistical analysis was performed using Meta-Disc (version 1.4, http://www.hrc.es/investigacion/metadisc.html) and Review Manager (Version 5.3. Copenhagen: The Nordic Cochrane Centre, The Cochrane Collaboration, 2014).

## Result

### Description of the studies included

**Fig. 1:**
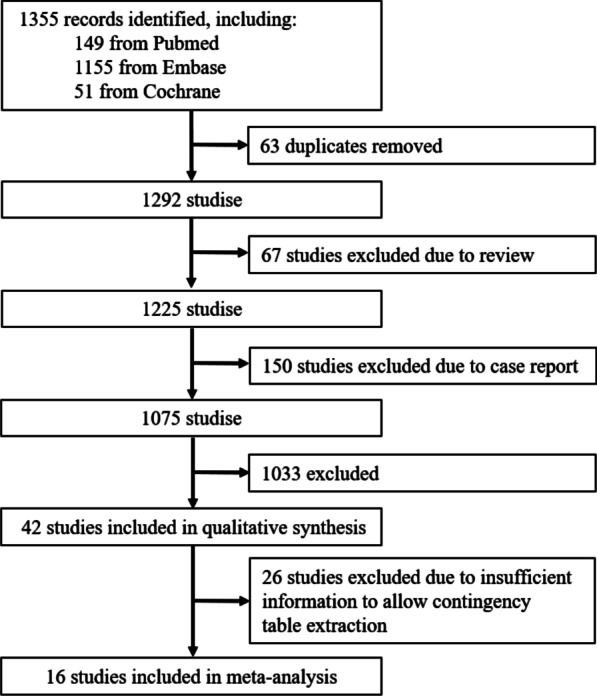
Workflow of study selection

A total of 1354 literatures were retrieved, including PubMed (n = 149), Embase (n = 1155) and Cochrane (n = 51). Among them, 63 duplicates, 67 reviews and 150 case reports were excluded, and 1033 studies that conform to the exclusion criteria were excluded. A total of 42 studies were included for literature quality assessment, and 26 of them were excluded due to lack of partial data. 16 articles were included for meta-analysis (Fig. 1).

In 16 articles, the main purpose of the five studies (31.25%, 5/16) were the polyp detection, and the image types used were mainly computed tomographic (CT) colonography. The other eleven studies (68.75%, 11/16) were mainly aimed at polyp classification, and the modalities used were narrow-band imaging (NBI), white light (WL) and methylene blue staining. Nine studies (56.25%, 9/16) compared the performance of AI with that of human doctors for polyp detection and classification. Among them, four studies (25.00%, 4/16) additionally compared the performance of doctors with different experiences for polyp classification. Only one study (6.25%, 1/16) presented externally validated results (External validation refers to independent data that is not used for model development but is used to evaluate model performance).

### Study characteristics

The studies were published between 2006 and 2020. All 16 studies reported the performance of AI model in diagnosing colon polyps, among them, 9 studies also compared the diagnostic performance of AI and endoscopic experts, and 4 studies compared the diagnostic performance of doctors with different seniority. Table 1 shows the detailed characteristics of the eligible studies. Table 2 shows the relevant inclusion and exclusion criteria for each included study. Table 3 shows detailed data on the performance of AI and/or humans in the diagnosis of polyps in each study.

**Table 2:** The inclusion and exclusion criteria for 16 included studies

Author	Inclusion criteria	Exclusion criteria
Halligan [24]	All patients were known to have colorectal polyps, proven by intraindividual, same-day total colonoscopy, which immediately followed computed tomographic colonography (CTC). The histological diagnosis of resected polyps is the gold standard	Patients with inherited polyposis syndromes were excluded, as were patients with cancer (because detection was to be focused on polyps)
Petrick [38]	NR	NR
Tischendorf [39]	A total of 641 patients underwent colonoscopy during a period of 5 months. The histological diagnosis is the gold standard	Patients with chronic inflammatory bowel disease, adenomatosis coli, coagulopathy, insufficient bowel preparation, or who had undergone colonoscopy within the last 3 years (except for patients referred for polypectomy of known polyps) were excluded from the study
Ignjatovic [7]	NR	NR
Gross [17]	The histological diagnosis is the gold standard	Chronic inflammatory bowel disease, adenomatosis coli, coagulopathy, insufficient bowel preparation, or previous colonoscopy within the past 3 years (except for patients who were sent for polypectomy of known polyps)
Mang [40]	All patients underwent computed tomographic colonography (CTC) and subsequent optical colonoscopy (OC) with histopathological work-up. Anonymised patient data were downloaded from a publicly accessible CTC training web site [46]	NR
Mesejo [23]	NR. The histological diagnosis is the gold standard. Videos with ground truth of this study are publicly available at http://www.depeca.uah.es/colonoscopydataset/	NR
Mori [41]	Between June and December 2017, serial patients scheduled for routine colonoscopy were 18 years or older, not receiving anticoagulant therapy, and able to give informed consent	A history of inflammatory bowel disease, chemotherapy, or radiation therapy for colorectal cancer
Renner [42]	Outpatients and inpatients aged ≥ 18 years scheduled for colonoscopy at the study site were eligible to participate. The histological diagnosis is the gold standard	Emergency examinations, Amercian Society of Anaesthesiologists risk classes IV and above, pregnant women, inflammatory bowel disease and polyposis syndromes
Chen [21]	From March 2017 to August 2017, polyps smaller than 5 mm from patients with an appropriate indication for colonoscopy. The histological diagnosis is the gold standard	Age younger than 18 years, fulminant colitis, severe hematochezia, and poor bowel preparation. The presence of a staining artifact created by mucus, out-of-focus and insufficiently bright images, motion-blurred images, and the presence of histologic features of sessile serrated adenoma/polyps
Shin [43]	NR. Data are available at three public datasets [47]	NR
Byrne [11]	The histological diagnosis is the gold standard	NR
Cristina [44]	The histological diagnosis is the gold standard	NR
Zachariah [35]	The histological diagnosis is the gold standard	NR
Shahidi [45]	From April 2016 to August 2017 consecutive colorectal lesions ≤ 3 mm, diagnosed during optical evaluation as adenatomous	A size restriction of ≤ 3 mm
Qadir [14]	NR	NR

NR, not reported

**Table 3:** Results of AI/human in diagnosis of polyps

Author	Years	Model type	AI or human	TP	FP	FN	TN
Halligan [24]	2006	CAD	AI	330	55	270	415
Petrick [38]	2008	CAD system	AI	51	44	33	112
Tischendorf [39]	2010	Linear classifier、k-NN、SVM	AI	305	42	15	56
Ignjatovic [7]	2011	NA	AI	274	49	41	266
Gross [17]	2011	SVM	AI	358	28	20	283
Mang [40]	2012	CTC CAD system	AI	580	44	44	212
Mesejo [23]	2016	RF、RS、SVM	AI	52	5	3	16
Mori [41]	2018	SVM	AI	2146	142	164	1272
Renner [42]	2018	CNN	AI	110	42	7	97
Chen [21]	2018	DNN-CAD	AI	181	21	7	75
Shin [43]	2018	SVM	AI	188	8	7	163
Byrne [11]	2019	CNN	AI	65	7	1	33
Cristina [44]	2019	SVM	AI	174	15	18	118
Zachariah [35]	2020	CNN	AI	6443	434	294	3971
Shahidi [45]	2020	CNN	AI	409	168	49	18
Qadir [14]	2020	Faster R-CNN	AI	8171	1166	1854	1347
Halligan [24]	2006	CAD	Expert	239	27	361	443
Petrick [38]	2008	CAD system	Expert	38	23	46	133
Tischendorf [39]	2010	Linear classifier、k-NN、SVM	Expert	305	21	15	77
Ignjatovic [7]	2011	NA	Expert	172	82	38	128
Ignjatovic [7]	2011	NA	Novice	70	44	35	61
Gross [17]	2011	SVM	Expert	699	46	57	576
Gross [17]	2011	SVM	Novice	632	69	124	553
Mesejo [23]	2016	RF、RS、SVM	Expert	46.2	6.7	8.7	14.2
Mesejo [23]	2016	RF、RS、SVM	Novice	49.3	10	5.7	11
Renner [42]	2018	CNN	Expert	188	46	36	216
Chen [21]	2018	DNN-CAD	Expert	367	55	9	137
Chen [21]	2018	DNN-CAD	Novice	671	95	81	289
Cristina [44]	2019	SVM	Expert	187	5	5	128

TP, true positive; FP, false positive; FN, false negative; TN, true negative; CAD, computer-aided diagnosis; K-NN, k-nearest neighbor; RF, random forests; RS, random subspaces; SVM, support vector machine; CNN, convolutional neural network; DNN-CAD, computer-aided diagnosis with a deep neural network

### Quality assessment

**Fig. 2:**
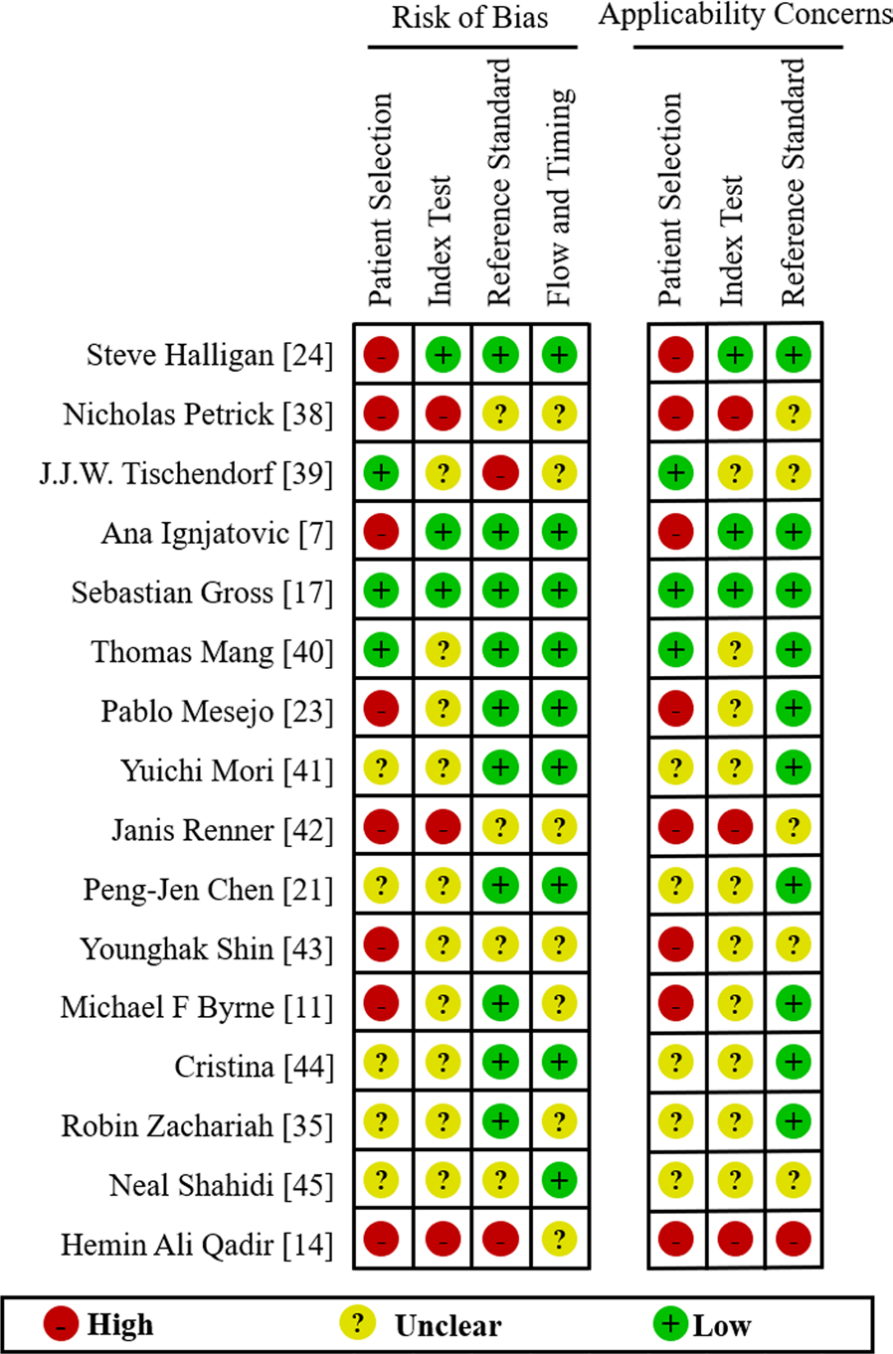
Quality Assessment of Diagnostic Accuracy Studies (QUADAS-2) risk of bias assessment. Review authors' judgements about each domain across included studies. Each row represents each included study. The columns consists of bias risks and applicability concerns. Red indicates high risk, yellow indicates unclear, and green indicates low risk

Study quality was assessed using QUADAS-2. Risk of bias and applicability concerns graph shows the authors' ratings of risk of bias and applicability concerns for each study (Fig. 2). For instance, data from some studies lacked detailed clinical information and the risk of bias in patient selection was rated as "unclear" or "high risk”.

### Diagnostic performance of AI/humans

**Fig. 3:**
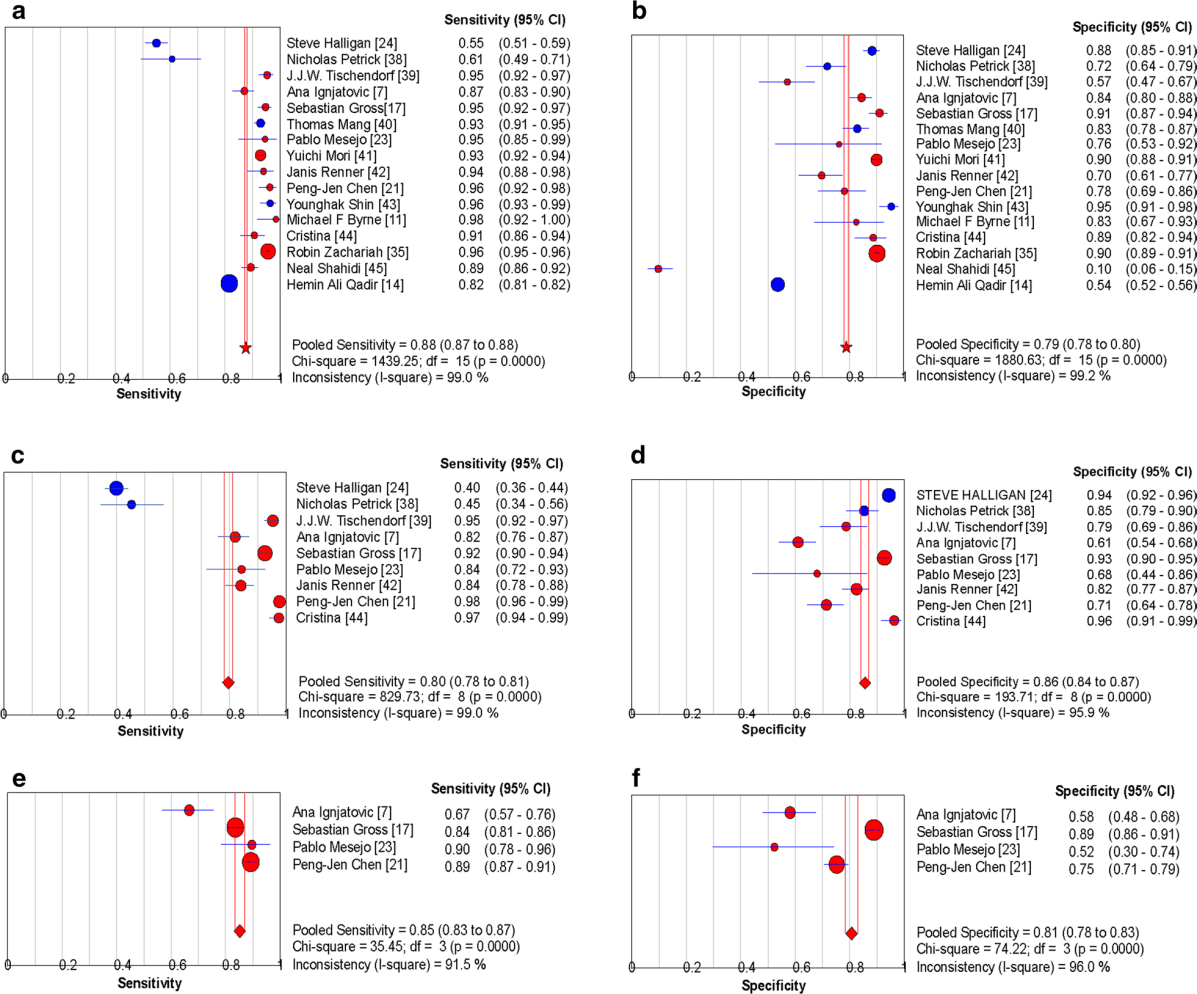
Forest plot of the sensitivity and specificity of AI and endoscopists in colon polyp detection and classification. **A** and **B** show the pooled sensitivity and specificity of AI for detection and classification of polyps. **C** and **D** show the pooled sensitivity and specificity of experts for detection and classification of polyps. **E** and **F** show the pooled sensitivity and specificity of non-experts for classification of polyps. The blue circle indicates that the main purpose of the article is the detection of colonic polyps, and the red circle indicates that the main purpose of the article is the classification of colonic polyps. The blue line in the figure shows the 95% confidence interval. The red star symbol represents pooled sensitivity and specificity. CI, confidence interval; DF, degrees of freedom

**Fig. 4:**
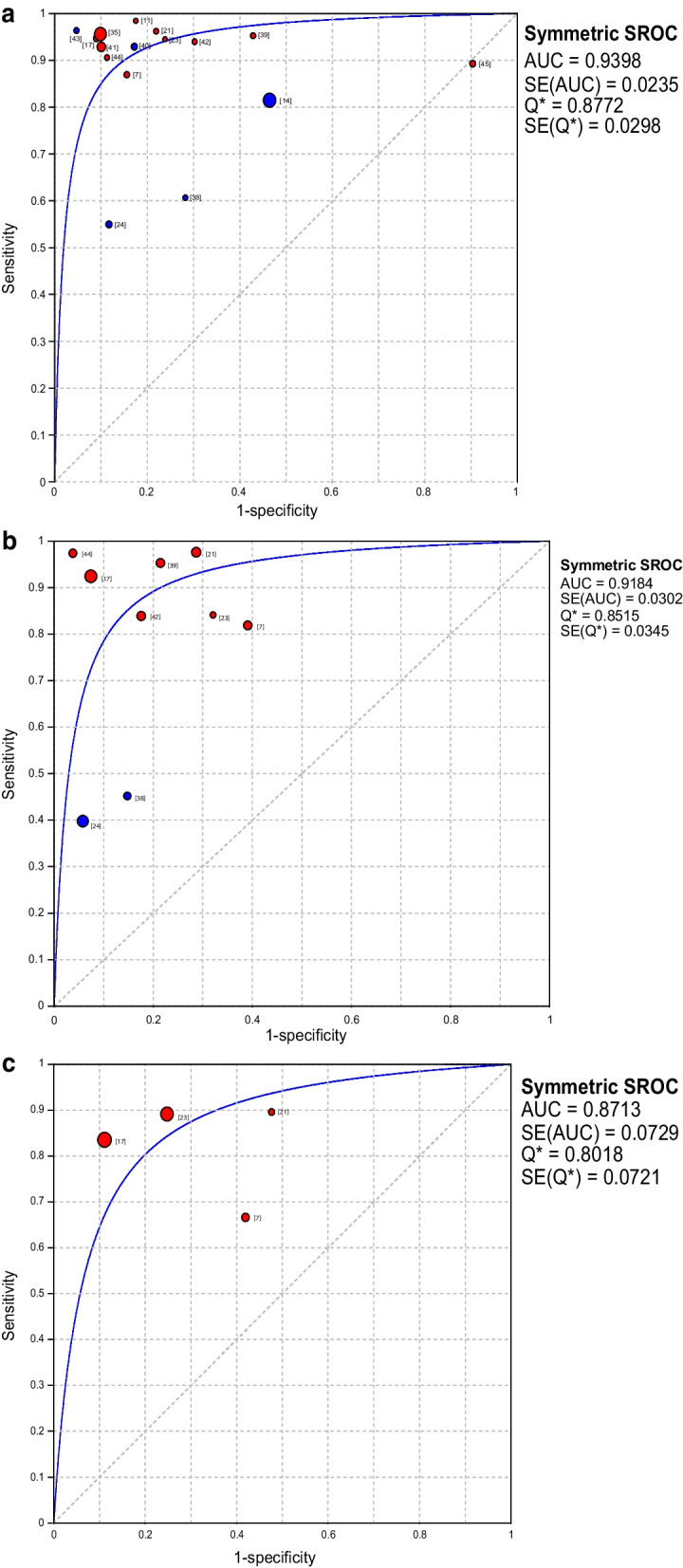
The summary receiver operating characteristic curve (sROC) for AI, expert and non-expert groups. **A** The sROC of AI for colon polyp detection and classification. **B** The sROC of experts for colon polyp detection and classification. **C** The sROC of non-experts for colon polyp classification. The blue circle indicates that the main purpose of the article is the detection of colonic polyps, and the red circle indicates that the main purpose of the article is the classification of colonic polyps. The size of the circle is proportionate to the number of patients enrolled for each study. AUC, area under the curve

A total of 16 studies used AI for polyp identification and diagnosis, and random effects models were used to estimate the effects. The pooled S_EN_ and pooled S_PE_ of AI in the diagnosis of polyps were 88% (95% CI 0.87–0.88) and 79% (95% CI 0.78–0.80), respectively (Fig. 3A, B). Figure 4A showed the sROC of AI for colon polyp detection and classification and the corresponding AUC was 0.940, and the Q index was estimated to be 0.877, indicating the excellent performance of AI in the detection and diagnosis of polyps. Spearman coefficient was − 0.282 (*P* = 0.289).

For the performance of endoscopic experts in polyp detection and diagnosis, a total of 9 studies included relevant data. The effects were estimated using the random effects model, with the pooled S_EN_ and pooled S_PE_ of 80% (95% CI 0.78–0.81) and 86% (95% CI 0.84–0.87) respectively (Fig. 3C, D). Figure 4B showed the sROC of experts for colon polyp detection and classification and the corresponding AUC was 0.918, and Q index was 0.852. Spearman coefficient was 0.050 (*P* = 0.898). Four of the studies included the diagnosis of polyps by doctors with less experience, with pooled S_EN_ and pooled S_PE_ of 85% (95% CI 0.83–0.87) and 81% (95% CI 0.78–0.83), respectively (Fig. 3E, F). Figure 4C showed the sROC of non-experts for colon polyp classification and the corresponding AUC and Q indexes were 0.871 and 0.802, respectively. Spearman coefficient was 0.400 (*P* = 0.600).

Threshold effect is due to studies published in different date and using different thresholds to define positive or negative, which results in the difference in S_EN_, S_PE_ or likelihood ratio between the studies. Threshold effect is one of the main causes of heterogeneity in experimental studies [28]. In this study, Spearman rank correlation coefficients were − 0.175 (*P* = 0.364), indicating no threshold effect.

### Compare traditional machine learning with deep learning

In this study, we also try to explore the comparison between traditional ML methods (such as Random forests model (RF), support vector machine (SVM), linear classifier, K neighbor, etc.) and DL (such as CNN) in the detection and classification of colonic polyps. Using meta-regression, the result shows that there is no significant difference between traditional machine learning and deep learning (*P* = 0.7989).

## Discussion

ADR of colon polyps is very important for the early diagnosis of colorectal cancer. Automatic detection of polyps based on colonoscopy can significantly increase the ADR, improve the detection rate of hyperplastic polyps, and reduce the rate of missed detection [29]. Artificial intelligence assisted systems are expected to improve the quality of automated polyp detection and classification [30]. It is only a matter of time before AI is used in the field of gastrointestinal endoscopy [15]. Liu et al. [31] conducted a meta-analysis of 82 studies on the comparison between deep learning and medical professionals, showing that AI has the same S_EN_ and S_PE_ as human beings.

The AUC under the sROC is an indicator to measure the reliability of the diagnostic method. The closer the AUC is to 1, the better the diagnostic effect is. In this study, the AUC of AI in polyp detection and classification was 0.940 (Fig. 4A), the AUC of the expert group and the non-expert group in polyp detection and classification were 0.918 and 0.871 (Fig. 4B, C), respectively. It can be seen that the performance of AI was similar to that of human experts, and higher than that of novice doctors. Lui et al. [12] conducted a systematic review of 18 studies comparing AI with human physicians in examining colon polyps. Their results showed that there was no significant difference in performance between the AI and the endoscopists, but the performance of AI was significantly better than that of the non-specialist endoscopists, which was similar to our conclusion. Based on the results of this study, we speculate that AI may could improve the performance of young doctors for detection and classification of colonic polyps. Some studies have found similar results [21, 32], however, it is still not clear how expertise is best transferred to community gastroenterologists and to trainees [7].

The pooled S_EN_s of AI, expert and non-expert were 88% (95% CI 87–88%), 80% (95% CI 78–81%), 85% (83–87%), respectively. Meanwhile, the pooled S_PE_s of AI, expert and non-expert were 79% (95% CI 78–80%), 86% (95% CI 84–87%), 81% (78–83%), respectively. From the research results, the AI group had slightly lower S_PE_ than the expert group (79% vs. 86%, *P* < 0.05), although the S_EN_ was higher than the expert group (88% vs. 80%, *P* < 0.05). The high S_EN_ of AI may suggest that in endoscopic screening, AI can better assist endoscopists in the discovery of polyps, improve ADR, and thus reduce the incidence and mortality of CRC. Interestingly, while the non-experts had less pooled S_PE_ in polyp recognition than the experts (81% vs. 86%, *P* < 0.05), they had higher pooled S_EN_ than the experts (85% vs. 80%, *P* < 0.05). We speculate that the reason for this phenomenon may be that when faced with some suspicious lesions, doctors with junior experience often do not have enough confidence to make judgments, so they uniformly judge them as polyps, resulting in high S_EN_ and low S_PE_. Of course, since only four of the included studies had data on junior physicians, care should be taken when interpreting these data.

Further, we performed a subgroup analysis of the included 16 papers according to the primary study task. The results revealed a relatively high specificity and low sensitivity in the studies with the primary aim of polyp detection (Figs. 3A–D, 4A, B). From the analysis of the results, we speculate that there may be several reasons for this phenomenon. First, since only 5 of the 16 included studies were on the task of polyp detection, there may be a case of data bias. Second, polyp detection and polyp classification are different tasks, resulting in different performance of the models. For the classification task, the model only needs to output the probability distribution of the category corresponding to the current overall image. While for the detection task, the model needs to output each polyp location and its classification probability for the whole image, which is a difficult challenge especially for the case where multiple polyps exist in a single image. Third, there are various polyp-like structures in the colon, and the size, color, shape and texture of polyps vary greatly between categories, making it very difficult to automatically detect polyps and sometimes miss the same polyps that appear in adjacent frames [14].

Different sensitivities and specificities can be obtained by setting corresponding thresholds according to the probability values output by the AI model in a particular task. The design of AI for colon polyp screening requires high sensitivity in primary care. In addition, a highly specific AI-assisted diagnostic system can also be designed for final diagnosis in secondary care. Our results show that AI can achieve higher sensitivity than humans while maintaining similar specificity, indicating the effectiveness advantage of AI, especially for primary care medical tasks such as colon polyp screening.

The results show that there is no significant difference between traditional machine learning and deep learning (*P* = 0.7989), which should be interpreted with caution due to the limitations of the included studies and their data. DL approaches differ significantly from traditional ML approaches in that they can extract features from raw data and learn them instead of using manual features based on feature engineering [33], which performs well in many tasks, including data denoising, target detection and classification [34].

Among the retrieved literatures, only one study [35] was externally validated, while the rest were internally validated only, which tended to lead to an optimistic evaluation of the model performance. Liu et al. [31] compared 82 studies on medical AI and found that only a few studies (25/82) provided external validation data, which is also similar to ours results. The model may have good performance in the internal data set, but it does not perform well in the new data set, and the generalization ability of the model is poor, which is not conducive to the universality of the model. In order to evaluate the performance of the prediction model more accurately, it is necessary to develop a new reporting standards on deep learning [36].

CNN is a deep neural network structure for image recognition, which has a very excellent ability [37]. Currently, most AI models, limited by hardware and data sets, are based on static images for lesion recognition. Of the included studies, only one used video training. Even though some studies claim to be able to detect in real time, they are based on the detection time of a single frame image, and can realize real-time monitoring in theory, but no practical clinical verification has been carried out. Therefore, in the future study, a model for video data can be developed and verified in clinical practice.

There are also some limitations in our analysis. Firstly, only one study (1/16) presented externally validated results, which is not conductive to the universality of the model. Secondly, the exclusion of reviews, conference papers, and letters may lead to publication bias, lack of consistency in reference criteria, duration of follow-up, and other important variables may affect the diagnosis. Thirdly, the included studies used different image modalities, which may have biased the results. Fourthly, the heterogeneity of the studies, which included large time spans, may lead to large differences in the observed performance of the AI model and endoscopic experts. We conducted a heterogeneity analysis of the study, and although Spearman coefficient (− 0.175) and sROC plots showed no threshold effect, different AI models may lead to threshold effect, resulting in heterogeneity. In this case, it may be necessary to limit the analysis to a subset of studies that share a common threshold. However, we did not perform this analysis because most studies did not provide detailed diagnostic thresholds.

## Conclusion

In conclusion, this meta-analysis demonstrated that, in general, AI has high sensitivity and moderate specificity for polyp detection and classification, similar to that of human experts, and can be used as an aid. The difference between polyp classification and polyp detection tasks, however, leads to differences in the performance of deep learning models and human experts for different tasks, especially for sensitivity and specificity, which suggests that the possible impact of different tasks on the models should be considered when building the models. In addition, the application of deep learning in colonoscopy needs more external validation. Limited by the sample size of data included in this meta-analysis, further studies are needed to evaluate it in the future.

## Data Availability

All data generated or analyzed during this study are included in this published article.
